# Effects of fungal infection on the survival of parasitic bat flies

**DOI:** 10.1186/s13071-020-3895-8

**Published:** 2020-01-13

**Authors:** Tamara Szentiványi, Péter Estók, Romain Pigeault, Philippe Christe, Olivier Glaizot

**Affiliations:** 1 Museum of Zoology, Palais de Rumine, Place de la Riponne 6, 1014 Lausanne, Switzerland; 2 Department of Ecology and Evolution, University of Lausanne, Biophore, 1015 Lausanne, Switzerland; 3 Department of Zoology, Eszterházy Károly University, Eszterházy tér 1, 3300 Eger, Hungary

**Keywords:** Bat, Ectoparasite, Fungal infection, Laboulbeniales, Lifespan, Nycteribiidae

## Abstract

**Background:**

Parasites are able to alter numerous aspects of their hosts’ life history, behaviour and distribution. One central question in parasitology is to determine the degree of impact that parasites have on their hosts. Laboulbeniales (Fungi: Ascomycota) are ectoparasitic fungi of arthropods. Even though these fungi are widely distributed, little is known about their ecology and their possible physiological effects on their hosts. We used a highly specific bat fly-fungi association to assess the effect of these fungal parasites on their dipteran hosts.

**Methods:**

We collected bat flies (Diptera: Nycteribiidae) belonging to two species, *Nycteribia schmidlii* and *Penicillidia conspicua* from their bat host *Miniopterus schreibersii* (Chiroptera: Miniopteridae). We experimentally tested the effect of infection on the lifespan of bat flies.

**Results:**

The prevalence of Laboulbeniales fungi was 17.9% in *N. schmidlii* and 64.8% in *P. conspicua*. Two fungi species were identified, *Arthrorhynchus eucampsipodae* and *A. nycteribiae*, both showing strict host specificity with *N. schmidlii* and *P. conspicua*, respectively. We found that fungal infection reduced by half the survival rate of *P. conspicua* regardless of sex, whereas *N. schmidlii* was not affected by the infection. Moreover, the intensity of infection showed negative correlation with the lifespan of *P. conspicua*.

**Conclusions:**

To our knowledge, this is the first indication that fungal infection can alter bat fly survival and thus may play a significant role in the population dynamics of these bat ectoparasites.
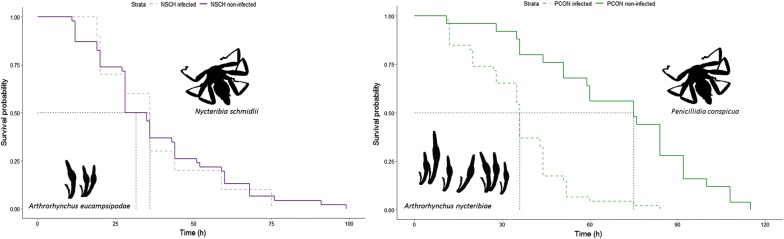

## Background

All animals harbour at least one, but most likely numerous parasites during their lifetime. The direct and indirect effects of parasites on their hosts is a widely studied subject in parasitology, ecology and evolution research. Parasites may broadly influence their hosts behaviour, physiology and life history traits such as survival or reproductive success [1–3]. For instance, the Bechsteinʼs bat (*Myotis bechsteinii*) avoids occupying roosts where bat fly puparia infection is high, which leads to a roost selection strategy to avoid being infected by newly emerged flies [4]. Host survival might be shortened with the increase of parasite pressure [5–7] and parasite infection may also have a strong influence on reproductive success, such as lower number of offsprings under high parasitic pressure [8–10]. Regarding host physiology, both negative or positive correlations between host body condition and parasite burden are found in several taxa [11–16].

With the notable exception of some entomopathogens such as *Metarhizium* or *Beauveria* [17–20], the costs of fungi infection on their invertebrate hosts are poorly documented. Parasitic fungi are frequently found across insect taxa, with nearly 1000 entomopathogenic species recognized to date [21]. Among them, the entomophagous, ectoparasitic Laboulbeniales species parasitize several arthropod orders such as Coleoptera, Diptera and Heteroptera, with about 80% of the known species occurring on beetles [22]. Relatively little is known about the physiological effect of Laboulbeniales infection on their hosts. Laboulbeniales-infected ants have been shown to have a reduced survival rate and/or to experience costly behavioural changes [23, 24]. Other effects, such as an increase in water consumption, have also been observed in Laboulbeniales-infected ants [23]. In the invasive lady beetle, *Harmonia axyridis* (Coleoptera: Coccinellidae), the survival rate of individuals was lower when infected [25]. Laboulbeniales distribution can be strongly altered by climatic and microclimatic factors in both ants and bat flies and thus differentially affects bat fly populations [26, 27].

Bat flies are obligate, hematophagous ectoparasites of bats, belonging to two families of the superfamily Hippoboscoidea, Nycteribiidae and Streblidae, along with Hippoboscidae (louse flies, ked flies) and Glossinidae (tsetse flies). Nycteribiidae have a higher diversity in the Old World, whereas Streblidae are mostly found in the New World, although both families have worldwide distributions. There are currently 16 species (and subspecies) of nycteribiids and one species of streblid known from Europe [28]. Bat flies show extreme morphological adaptations to their bat hosts, such as loss of wings (Nycteribiidae), reduced or missing eyes, and dorsoventrally (Nycteribiidae and Streblidae) or laterally (Streblidae) compressed body shape [29]. The reproductive biology of bat flies is rather unique. One single larva develops inside the female body, feeding on the secretum of the so-called milk glands. The larva has its first two larval stages within the female, which will be laid when reaches the third-instar larval stage. The larva immediately pupates and the imago emerges 3 to 4 weeks later, before actively searching for a host [30, 31]. Females live about 1.5 times longer than males. The total adult lifespan of *Basilia hispida* is on average 97 days for males and 156 days for females [31]. Unfed, newly emerged bat flies resist starvation up to 1–3 days, although adults which already fed at least once usually die within a day [32]. Bat flies are vectors of the haemosporidian *Polychromophilus* spp., a blood parasite closely related to *Plasmodium*, as well as suspected vectors of other pathogens such as the gram-negative bacteria *Bartonella* spp. [33–35]. Little is known about the interaction between pathogens and bat flies. Nevertheless, it has been previously shown that bat fly survival is decreased when infected with *Polychromophilus murinus* [36].

The aim of this study was to experimentally investigate the cost of Laboulbeniales infections on the survival of nycteribiid bat flies. Such costs may affect the population dynamics of the ectoparasitic flies but also the infection dynamics of vector-borne parasites transmitted by bat flies [37].

## Methods

Bats were captured in September 2016, 2017 and 2018 at various sites in Hungary (Additional file 1: Table S1). Bat flies were collected from the common bent-winged bat, *Miniopterus schreibersii* (Chiroptera: Miniopteridae). Immediately after collection, flies were individually placed into separate tubes and kept at 4 °C, in order to prolong their lifespan [36]. Flies were examined every 8 hours to see any sign of life, as movement of body parts. The experiment finished when all collected flies were dead. Dead flies were stored in 98% ethanol for further identification, based on Theodorʼs key [38]. Laboulbeniales species were identified using several keys [39, 40]. The presence of Laboulbeniales and the intensity of fungi infection, defined as the number of mature thalli found on an infected host were recorded using a stereomicroscope (Leica M205C, Leica Microsystems AG, Heerbrugg, Switzerland). Prevalence was assessed as the proportion of infected individuals within the host population, according to previously defined parasitological terms [41].

Prevalence and intensity were analysed using a linear mixed-effects model procedure with binomial or normal errors respectively (package *lme4* in R [42]). We first tested whether infection prevalence and intensity varied according to fly species. Then we investigated the impact of sex on both variables within each species. Collection year was used as a fixed factor and location was fitted as a random variable in the models.

Lifespan was calculated as the number of survived hours between collection of flies and observation of death. Survival was analysed using Cox proportional hazards regression model (CoxPHM), which calculates the hazard ratio by estimating the difference of relative risk of death between different groups (hr_present/absent_) [43], using the *survival* package in R [44]. In order to test the effect of sex, infection presence and intensity on lifespan, we used linear mixed-effects model with normal error distribution. In the models, we included the lifespan (h) as the response variable, whereas bat fly sex, presence/absence of infection or intensity of infection and collection year were used as fixed factors. Location was fitted as a random variable in the models. Full models, including all explanatory variables and the interactions, were simplified by sequentially eliminating non-significant interactions and terms to establish a minimal model [45]. The significance of the explanatory variables was established using a likelihood ratio test (which approximately follows a Chi-square distribution [46]). The significant Chi-square given in the text are for the minimal model, whereas non-significant values correspond to those obtained before the deletion of the variable from the model. Statistical analyses were performed using R 3.5.3 [47].

## Results

### Prevalence and thallus intensity

A total of 127 bat flies were collected (Table 1). *Nycteribia schmidlii* were exclusively infected by *Arthrorhynchus eucampsipodae*, whereas *Penicillidia conspicua* were infected by *A. nycteribiae*. The infection prevalence by Laboulbeniales fungi was higher in *P. conspicua* (64.8%) than in *N. schmidlii* (17.9%) ($\chi^{ 2}_{ 1}$ = 30.343, *P* < 0.0001). No effect of sex on infection prevalence was observed in both species (*N. schmidlii*: $\chi^{ 2}_{ 1}$ = 0.026, *P* = 0.871; *P. conspicua*: $\chi^{ 2}_{ 1}$ = 1.836, *P* = 0.175). Infection prevalence was however impacted by the year of capture in *P. conspicua*, but not in *N. schmidlii* ($\chi^{ 2}_{ 1}$ = 8.339, *P* = 0.015 and $\chi^{ 2}_{ 1}$ = 2.527, p = 0.287, respectively). Infection prevalence was higher in *P. conspicua* collected in 2016 (100%) than in 2017 (59%) and 2018 (58%).

**Table 1:** Number of female and male infected (collected) bat flies *Nycteribia schmidlii* and *Penicillidia conspicua* by the Laboulbeniales fungi *Arthrorhynchus eucampsipodae* and *A. nycteribiae* as well as prevalence and mean intensity ± SE (number of thalli per individual)

	Female	Male	Total	Prevalence (%)	Mean intensity
*N. schmidlii*	8 (42)	2 (14)	10 (56)	17.9	6.8 ± 1.8
*P. conspicua*	27 (38)	19 (33)	46 (71)	64.8	55.4 ± 8.9

Thalli intensity (number of thalli/individual) follows a negative binomial distribution in both species (*N. schmidlii*: variance-to-mean ratio = 4.67; *P. conspicua*: variance-to-mean ratio = 65.49). The highest intensity was 251 thalli on a single *P. conspicua*. The mean thalli intensity (excluding non-infected individuals) was approximately eight times higher in *P. conspicua* (55.4 ± 8.9 thalli per individual) compared to *N. schmidlii* (6.8 ± 1.8, $\chi^{ 2}_{ 1}$ = 19.619, *P* < 0.0001). An effect of sex on intensity was observed only in *P. conspicua* (*P. conspicua*: $\chi^{ 2}_{ 1}$ = 4.494, *P* = 0.034; *N. schmidlii*: $\chi^{ 2}_{ 1}$ = 0.477, *P* = 0.489). The number of thalli was more than twice as high in infected *P. conspicua* females than in males (females: 72.48 ± 12.72; males: 31.10 ± 9.46). The intensity of infection measured in both species was not impacted by the year of capture (*P. conspicua*: $\chi^{ 2}_{ 1}$ = 0.556, *P* = 0.757; *N. schmidlii*: $\chi^{ 2}_{ 1}$ = 2.527, *P* = 0.283).

### Distribution of infection on host body parts

Fungal thalli were found on the head or the dorsal part of thorax and abdomen of *N. schmidlii* whereas most of the thalli were observable on both dorsal and ventral part of the abdomen of *P. conspicua*, although all body parts showed signs of infection in many cases (Table 2).

**Table 2:** Total number of infections observed on different body parts of bat flies (F/M: on females and males respectively) for both species *N. schmidlii* and *P. conspicua*

Infected body part	*N. schmidlii* (F/M)	*P. conspicua* (F/M)	Total
Legs	0	11 (8/3)	11
Head	1 (1/0)	5 (4/1)	6
Thorax (dorsal)	7 (5/2)	4 (2/2)	11
Thorax (ventral)	0	1 (0/1)	1
Abdomen (dorsal)	4 (4/0)	31 (23/8)	35
Abdomen (ventral)	0	31 (19/12)	31
Genitalia	0	5 (0/5)	5

### Infection and survival rate

**Fig. 1:**
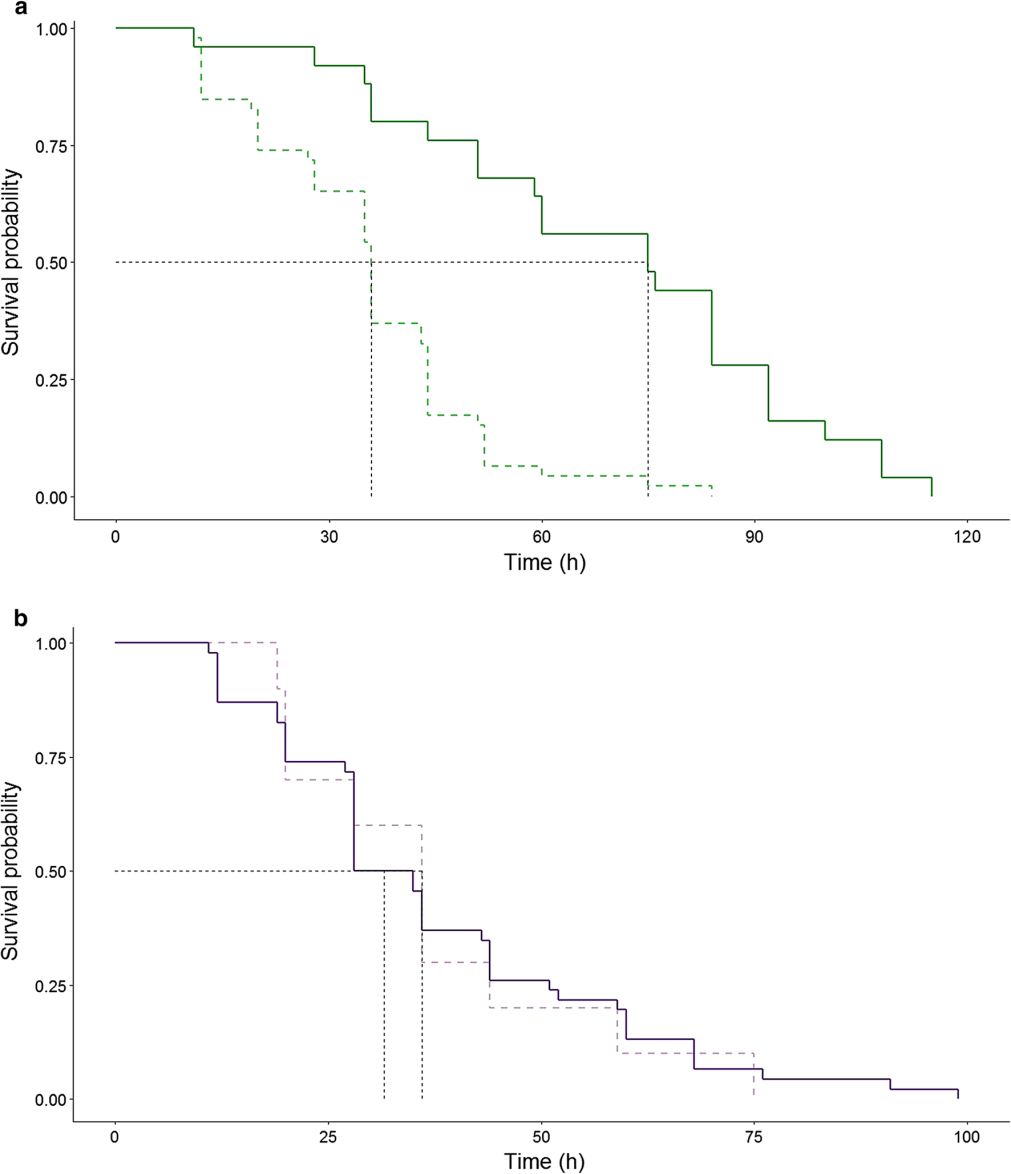
Survival curves of bat flies, *Penicillidia conspicua* (**a**, green) and *Nycteribia schmidlii* (**b**, purple). Solid lines are uninfected individuals, dashed lines are infected ones. Survival median lines are indicated with black dashed lines

*Penicillidia conspicua* individuals infected with *A. nycteribiae* (*n* = 46) had lower survival rate than non-infected individuals (*n* = 25) (Fig. 1a, *χ*^2^ = 30.08, *P* < 0.0001). The risk of death in infected individuals was 80% higher than of non-infected individuals in *P. conspicua* (hr = 0.2). However, the survival rate of *N. schmidlii* infected with *A. eucampsipodae* did not differ between infected (*n* = 10) and non-infected (*n* = 46) (Fig. 1b, hr = 0.93, *χ*^2^ = 0.04, *P* = 0.84) individuals.

### Impact of infection and sex on *Penicillidia conspicua* lifespan

**Fig. 2:**
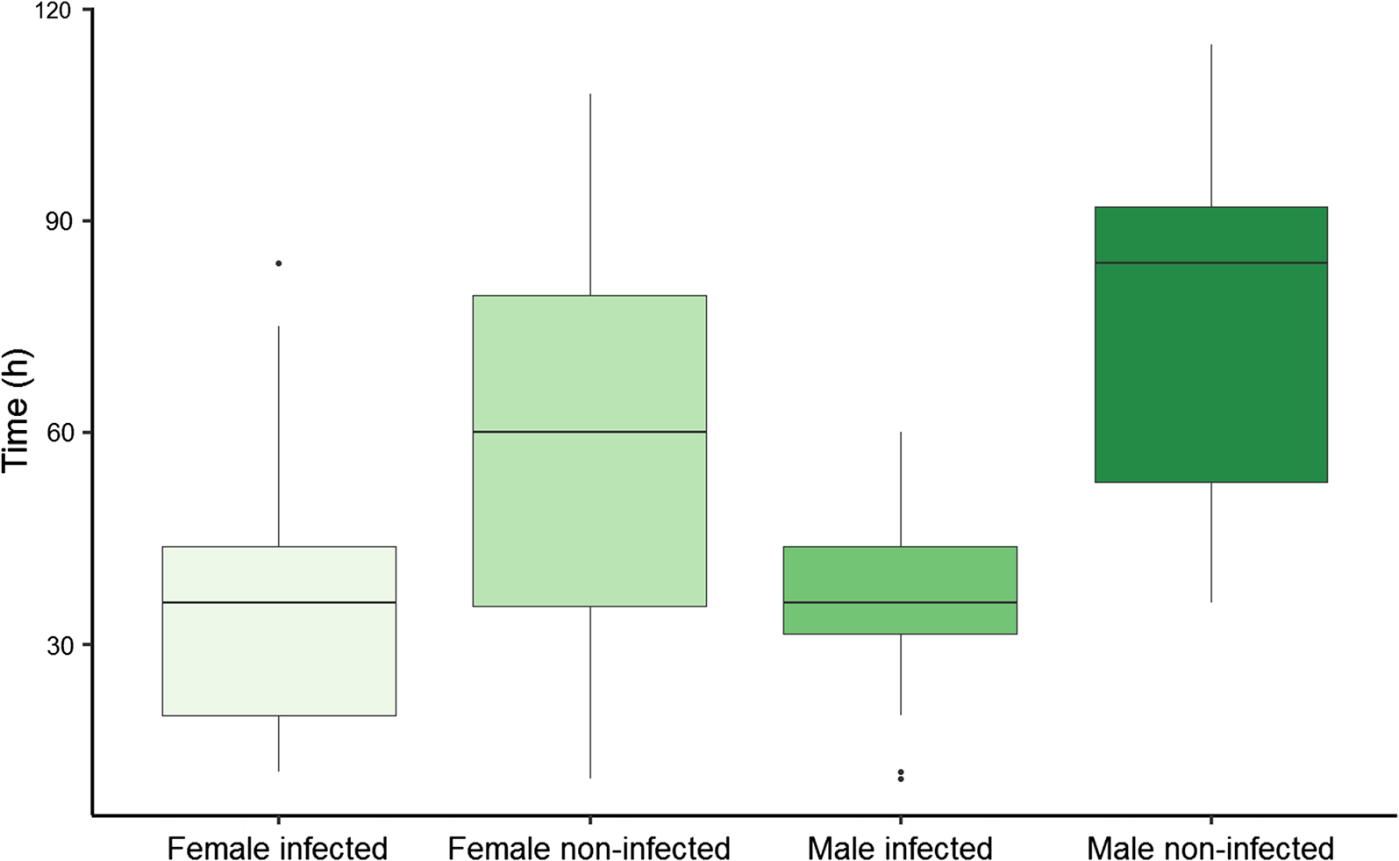
Lifespan (hours) of infected and non-infected females and males *Penicillidia conspicua* under experimental conditions

**Fig. 3:**
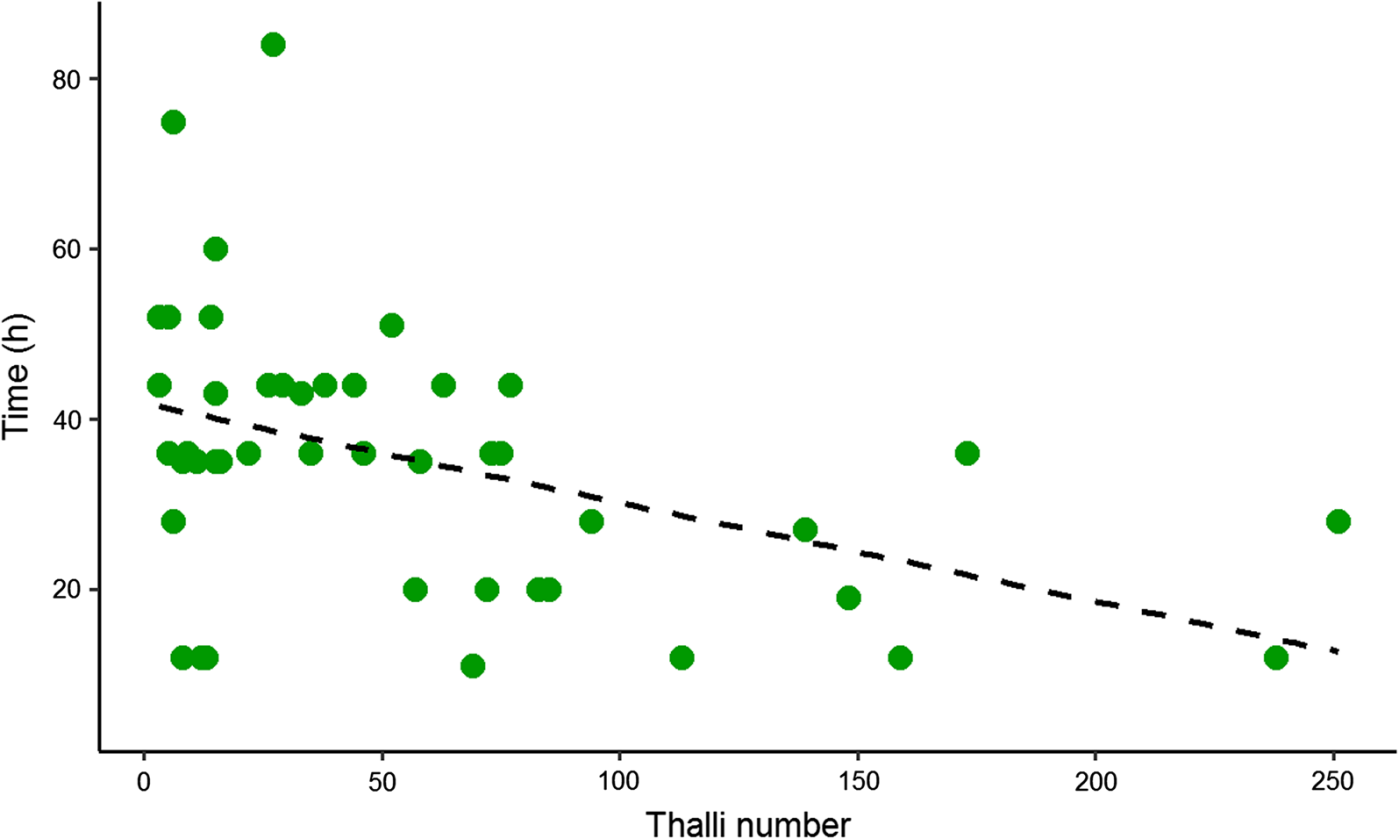
Lifespan (hours) of infected *Penicillidia conspicua* as a function of the intensity of infection (number of thalli) under experimental conditions

Fungi infection had a significant effect on *P. conspicua* lifespan (mean lifespan 47.4 ± 3.2; uninfected: 69.6 ± 5.6; infected: 35.4 ± 2.4; $\chi^{ 2}_{ 1}$ = 28.5, *P* < 0.005). The year of capture ($\chi^{ 2}_{ 1}$ = 0.01, *P* = 0.89) or sex had no effect on host lifespan (mean lifespan females: 43.1 ± 4 h; males 52.5 ± 4.8 h; $\chi^{ 2}_{ 1}$ = 0.51, *P* = 0.47, Fig. 2). When we focused on infected individuals only, a strong effect of the infection intensity (number of thalli) was observed on *P. conspicua* lifespan ($\chi^{ 2}_{ 1}$ = 10.7, *P* < 0.001, Fig. 3).

## Discussion

Similarly to previous studies [39, 48], we confirm the high specificity of the two fungal parasites, *A. eucampsipodae* and *A. nycteribiae*, towards *N. schmidlii* and *P. conspicua*, respectively. Infection prevalence and intensity strongly differed depending on the fly/fungi associations. In addition, these two species of parasites did not have the same effect on the survival of their host.

Fungi infection significantly shortens the survival of *P. conspicua*, but not of *N. schmidlii*. Such effects of Laboulbeniales on host survival have been observed in several distantly related taxa, such as ants and beetles [23, 25, 49, 50]. In our study, we found that infected *N. schmidlii* bears 6.8 thalli on average, whereas the average for *P. conspicua* was 55.4. This may suggest an effect of the intensity of infection on lifespan, although the different fungi species might use different strategy of host exploitation. It was recently documented that *A. nycteribiae* penetrates the host cuticle, most likely to extract nutrients [51], which possibly has a strong impact on the survival of their bat fly hosts. The amount of resource used by the fungus, and thus the costs induced by infection, increases with parasite burden. Further costly changes include that infection presence might reduce mating and reproductive success [9]. Additionally, the weight of the parasitic fungi itself might be costly to their bat fly hosts by increasing physiological energy consumption and thus may shorten lifespan.

In our experiments, we had no control of the age of the fly in the different experimental groups and it might be argued that older flies were more likely to be infected, inducing a confounding effect of age in our results. Characters which are normally used in age determination of insects [52] are however missing or not possible to obtain in bat flies. In ants, Laboulbeniales infection intensity increased with host age [24, 53], which can be the result of behavioural differences between younger and older individuals, and/or effect of general lifespan: older individuals have more time to obtain infection [53]. In addition, an immune senescence is observed in some invertebrate species [54, 55]. Nonetheless, in several invertebrate species older individuals are more resistant to parasite infection compared to young ones [56]. Only experimental infection of individuals of known age would permit to investigate the impact of the interaction between age and infection on the lifespan of bat flies.

Sex-biased Laboulbeniales infection might occur in different host taxa but can vary seasonally and with fungi species [57–59]. Either no strong sex bias or occasional male biased infection is found in *H. axyridis* [57, 58, 60]. Previous studies more often found female biased infection in bat flies [39, 48]. Our results with *P. conspicua* show that prevalence was not affected by the sex of the fly and that both sexes had shortened lifespan when infected. Nevertheless, the intensity of infection was more than twice as high in infected *P. conspicua* females compared to males.

Position specificity (infection occurrence on certain body parts) in Laboulbeniales have been observed in multiple studies among wide variety of host taxa. Position specificity might change depending on host species [61], host sex [59] and mating behaviour [58, 62, 63], but also seasonally [58] or based on the intensity of infection [27]. Fungi thalli dominantly occur on the head, abdomen and thorax (but can be present on any body parts) of infected *Myrmica scabrinodis* (Hymenoptera: Formicidae), however it can vary based on the intensity of infection [27]. In *H. axyridis*, the distribution of the thalli on abdomen reflects the sexual behavior of the species during mating period. In contrast, during hibernation, thalli infection exhibits the aggregation behaviour of the individuals and shifts to the head and to the legs [58]. Similarly, there is a strong indication that infection is transmitted during copulation in millipedes, based on thalli distribution [63]. Here we also found evidence of body part specificity of *A. nycteribiae*, which might be likewise the result of mating behaviour in *P. conspicua*. Females are most likely infected on the dorsal part of the abdomen, whereas males occur to be infected most frequently on the ventral (and dorsal) part of abdomen and genitalia. This pattern can be explained by the position of the flies during copulation and indicates that infection is most likely transmitted during direct body contact between individuals. Such pattern was not found in *N. schmidlii*, nonetheless, only 10 infected individuals were collected, and larger sample size is needed to have a better understanding of *A. eucampsipodae* distribution on host body parts.

The presence of Laboulbeniales infection may increase host survival in ants, through mediating anti-pathogen protection against other fungi, such as the entomopathogen *Metarhizium brunneum* (Ascomycota: Hypocreales) [64]. Yet, the presence of Laboulbeniales infection leads to increased expression of immune genes involved in wound repair, suggesting that the fungal presence itself can be costly [64]. To our knowledge, there are no records of entomopathogenic fungi infecting bat flies besides Laboulbeniales and interaction with other pathogenic fungus is unlikely. Although streblid bat fly pupae are commonly observed to be infected by certain fungus, which might cause pupal mortality [65], observations of Laboulbeniales infection on pupae are still missing. However, since bat flies are associated with a wide range of microparasitic organisms [66], Laboulbeniales may have interaction with other parasites. As bat flies are vectors of bat malaria-like parasites, Laboulbeniales infection might affect vectorial capacity. For instance, in mosquitoes, *Penicillium* infection has been observed to increase susceptibility to *Plasmodium* infection [67] which in turn can reduce the survival of the vector [68]. In addition, the presence of *Polychromophilus* infection is known to reduce bat fly lifespan [36], although not investigated in this study, it might also have additional interaction with Laboulbeniales infection. This possible interaction between vectorial potential and fungi infection in bat flies needs to be explored in future studies.

## Conclusions

Bat flies are common ectoparasites of bats and they contribute to the maintenance and transmission of vector-borne pathogens in bat populations (e.g. *Polychromophilus* spp.). It is essential to recognize the complex interactions that shape the population dynamics of bat flies, in order to understand the factors that may affect vector populations. Laboulbeniales infection is relatively common on the highly specific bat flies of the cave-dwelling bat, *Miniopterus schreibersii*. Beforehand this work, we had no information about the possible physiological effects of this fungal infection on the bat fly hosts. We found that fungal infection does negatively affect the lifespan of the bat fly host, *P. conspicua*. Additionally, intensity of infection negatively correlates with the survival of these flies. Host sex does not seem to affect survival time under infection. To our knowledge, this study provides the first evidence that fungal infection has a negative effect on the survival of parasitic bat flies suggesting that Laboulbeniales may alter the population dynamics of bat flies under natural conditions.

## Supplementary information

**Additional file 1: Table S1.** Collection data (sex, location, date and infection status) and survival time of *Nycteribia schmidlii* and *Penicillidia conspicua.*

## Data Availability

The dataset supporting the conclusions of this article is included in Additional file 1: Table S1.
